# Application of the anti-IgLON5 disease composite score to assess severity, clinical course, and mortality in a French cohort

**DOI:** 10.1007/s00415-025-13001-7

**Published:** 2025-03-19

**Authors:** Antonio Farina, Macarena Villagrán-García, Amna Abichou-Klich, Marie Benaiteau, Emilien Bernard, Lucia Campetella, Florent Cluse, Virginie Desestret, Pauline Dumez, Nicole Fabien, David Goncalves, Sergio Muñiz-Castrillo, Géraldine Picard, Anne-Laurie Pinto, Véronique Rogemond, Alberto Vogrig, Bastien Joubert, Jérôme Honnorat

**Affiliations:** 1 French Reference Centre on Paraneoplastic Neurological Syndromes and Autoimmune Encephalitis, Hospices Civils de Lyon, Hôpital Neurologique, 59 Boulevard Pinel, 69677 Bron Cedex, France; 2 MeLiS-UCBL-CNRS UMR 5284, INSERM U1314, Université Claude Bernard Lyon 1, Lyon, France; 3 Service de Biostatistique et Bioinformatique, Hospices Civils de Lyon, Pôle Santé Publique, Lyon, France; 4 French Reference Centre on Amyotrophic Lateral Sclerosis, Hospices Civils de Lyon, Hôpital Neurologique, Bron, France; 5 Electroneuromyography and Neuromuscular Diseases Unit, Hospices Civils de Lyon, Hôpital Neurologique, Bron, France; 6 Service d’Immunologie, Centre de Biologie et Pathologie Sud, Hospices Civils de Lyon, Pierre-Bénite, France; 7 Department of Neurology, Hospital Universitario, 12 de Octubre, Madrid, Spain; 8 Department of Medicine (DMED), University of Udine, Udine, Italy; 9 Clinical Neurology, Department of Head-Neck and Neuroscience, Azienda Sanitaria Universitaria Friuli Centrale (ASUFC), Udine, Italy

**Keywords:** Anti-IgLON5 disease, Anti-IgLON5 antibodies, Neuronal surface antigen, Autoimmune encephalitis

## Abstract

Anti-IgLON5 disease presents with diverse symptoms, whose severity can be measured by the anti-IgLON5 disease composite score (ICS). This study applied the ICS to a retrospective anti-IgLON5 disease cohort (*n* = 52; median age 72 years, 63% male) diagnosed in the French Reference Center on Autoimmune Encephalitis (2016–2024), aiming to describe severity and clinical course, and to assess its potential to predict mortality. At diagnosis, the ICS distribution (median 18) aligned with previous publications and correlated with the time to diagnosis (median 19 months); all patients had symptoms in ≥ 2 ICS domains: bulbar (88%), sleep (84%), movement disorders (90%), cognition (64%), and/or other (78%). Of 46 patients with follow-up data, 7 (16%) died shortly after diagnosis; for the others, changes in the ICS mirrored the clinical course: at last visit, it decreased in improving patients (16/46, 35%; median 12 vs 17; *p* = 0.004), increased in worsening patients (11/39, 24%; median 26 vs 21; *p* = 0.006) and did not change significantly in stable patients (12/46, 26%; median 16 vs 15; *p* = 0.222). In the ROC analyses, 2-year mortality was predicted by the total ICS at diagnosis (AUC 69.51, 95% CI [50.19; 88.83]; optimal cut-off > 20, sensitivity 59%, specificity 77%), and by the bulbar score at diagnosis (AUC 74.68, 95% CI [56.17, 93.19]; optimal cut-off > 3, sensitivity 83%, specificity 62%). The ICS is a reproducible tool for assessing anti-IgLON5 disease severity and clinical course. Higher total and bulbar ICS at diagnosis are associated with increased mortality risk, underscoring the need for early and intensive management of bulbar dysfunction.

**Supplementary Information:**

The online version contains supplementary material available at 10.1007/s00415-025-13001-7.

## Introduction

Anti-IgLON5 disease is a recently characterized neurological disorder associated with antibodies targeting IgLON5 (IgLON5-Abs), a neuronal surface protein of unknown function [1, 2]. Even if initial autopsy studies found a novel neuronal tauopathy [1], several observations suggest that the disorder is primarily autoimmune, with neurodegeneration occurring as a late event. These observations include the pathogenic effects of IgLON5-Abs in vitro [3–5], a strong association with HLA-DQB1*05 subtypes [6], the absence of tau deposits in brain specimens of patients with short disease duration [7], inflammatory changes in the cerebrospinal fluid (CSF) of patients sampled early after onset [8, 9], and a higher likelihood of improvement in patients receiving early immune treatments [8].

However, most patients with anti-IgLON5 disease are diagnosed after a prolonged interval (usually more than 1 year) from clinical onset [8, 10]. This delay is likely due to its heterogeneous presentation, which includes a variety of symptoms such as sleep disorders, bulbar dysfunction, gait and movement abnormalities, and cognitive impairment [2, 8, 10, 11]. These symptoms often overlap and evolve progressively, similarly to neurodegenerative disorders [2, 8, 10, 11]. The impact of these symptoms on patients' functionality is not specifically measured by the modified Rankin scale (mRS), used in previous clinical studies to assess the severity and outcome of anti-IgLON5 disease [8, 10, 11]. To address this issue, the anti-IgLON5 disease composite score (ICS), covering the main clinical manifestations of the disease grouped into five domains (bulbar, sleep, movement disorders, cognition, and other symptoms), has recently been developed and tested in previously published nationwide cohorts from Spain (Barcelona cohort) and Germany (GENERATE cohort) [12]. In the present study, we applied the ICS to an independent, previously unpublished anti-IgLON5 disease nationwide cohort from France. Our aim was to assess the reproducibility of the ICS in describing disease severity at diagnosis and its clinical course, as well as to explore the potential role of the ICS and its partial scores as predictors of mortality.

## Materials and methods

### Patient inclusion and study design

All patients with serum and/or CSF samples positive for IgLON5-Abs at the French Reference Center on Autoimmune Encephalitis between February 1st, 2016, and March 31st, 2024, were identified. IgLON5-Abs were tested by immunofluorescence on rat brain slices and cell-based assay (HEK293 cells) as previously described [13]. Clinical and paraclinical data (brain magnetic resonance imaging [MRI] and CSF findings) were retrospectively extracted from all the available medical charts, and the treating physicians were contacted in case of missing information. Patients with no clinical data or alternative explanations for their neurological syndrome were excluded. Clinical onset was classified as subacute when substantial disability was reached in < 4 months, and otherwise as chronic [10]. The symptoms and their severity were classified according to the ICS [12], which was retrospectively evaluated at the time of diagnosis and at last visit. Since death is not graded in the ICS, for patients who had died by the end of the study period, the ICS was re-scored at last alive visit before death. Whenever possible, data from polysomnography and cognitive scales (i.e., Montreal Cognitive Assessment scale) were used to score the severity of sleep disorders and cognitive domains [12]. Non-evaluable symptoms were recorded as missing and scored as 0, and patients with > 3 missing symptoms were excluded from the ICS assessment [12]. The ICS at diagnosis was compared with the mRS at diagnosis, which was also retrospectively assessed [12]. The clinical course was classified based on the treating physicians' subjective and overall assessment during follow-up, as reported in the medical records. Patients were categorized as: improving, for those who showed an improvement in autonomy, quality of life, and/or neurological symptoms and signs; stable, for those without any substantial changes in these factors; and worsening, for those who experienced a decline in autonomy, quality of life, and/or neurological symptoms and signs. Additionally, patients who died shortly after the diagnosis of anti-IgLON5 disease were classified as having a fulminant course. The cut-offs to define elevated CSF cell counts and protein levels were > 5 cells/mL and > 0.45 g/L, respectively.

### Statistical analysis

Categorical variables were described by the frequency and percentage for each category; continuous variables were described by median, interquartile range (IQR) and/or range. Comparisons of the variables according to defined groups were performed using Fisher’s exact test for categorical variables, and non-parametric Wilcoxon–Mann–Whitney test for continuous variables. For multiple comparisons according to the disease course, the false discovery rate correction was applied. A Spearman’s rank correlation coefficient was estimated to assess the correlations between the ICS and the variables mRS at diagnosis, age at diagnosis, and time to diagnosis. A logistic regression analysis was performed to assess the effect of the administration of first- and second-line immunotherapies within the first year of clinical onset on the type of clinical course (dichotomized into two groups: improving and stable versus worsening), considered as the dependent variable. Odds ratios and Wald 95% confidence intervals were reported. To evaluate the ability of the total and partial ICS to predict the 2-year mortality, the empirical area under the curve (AUC) of time-dependent Receiver Operating Characteristic (ROC) analysis [14] was estimated with 95% confidence interval based on the Delong method [15]. The optimal cut-off value of each ICS score was defined as the one that maximized the modified Youden index [16]: the sum of sensitivity and specificity weighted by the observed mortality rate, fixed at 38%, and the relative cost of a false negative classification, fixed at 2. For this optimal cut-off, the sensitivity and specificity values were presented. The Kaplan–Meier curves were presented on the subgroup of ICS according to the optimal cut-off and were compared by the log-rank test. A Cox regression model adjusted by age was performed to assess the impact of the total and partial ICS on the 2-year mortality risk. Hazard ratios and its 95% confidence intervals were reported. Schoenfeld residuals were assessed to verify the proportional hazards assumption for each covariate.

Statistical analyses were performed using R, version 3.4.0 (R Foundation for Statistical Computing, Vienna, Austria). All *p* values were two-tailed, and *p* values < 0.050 were considered statistically significant.

## Results

### Cohort description

**Fig. 1:**
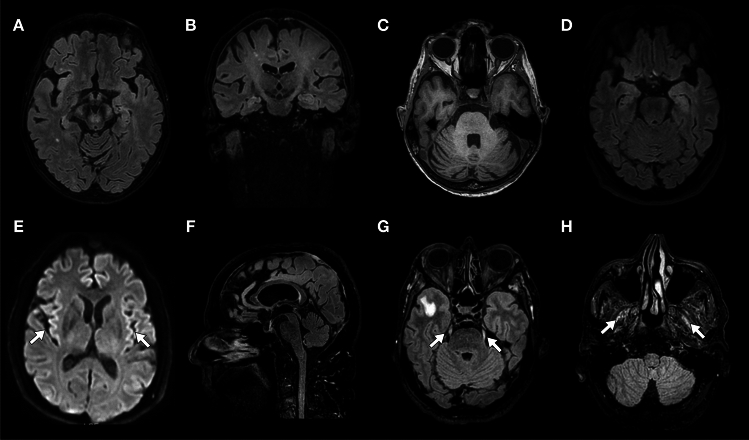
Brain magnetic resonance imaging findings. Mesencephalon (**A**) and pyramidal tracts (**A**, **B**) T2/fluid attenuated inversion recovery (FLAIR) hyperintensity, and pontocerebellar atrophy (**C**; case n. 47). Left medial temporal lobe T2/FLAIR hyperintensity (**D**; case n. 16). Diffusion-weighted imaging cortical hyperintensity (**E**, arrows; case n. 6). Mesencephalic atrophy (**F**; case n. 5). T2/FLAIR hyperintensity of the trigeminal nerves (**G**, arrows) and lateral pterygoid muscles (**H**, arrows; case n. 4)

We identified 57 patients positive for IgLON5-Abs in the French Reference Center. After exclusion of patients without clinical data (*n* = 3) or with alternative diagnoses (meningeal carcinomatosis, *n* = 2), 52 patients with anti-IgLON5 disease were included in the study (Supplementary Fig. 1). The median age at IgLON5-Abs detection was 72 years (IQR 68–78; range 51–86) and 33/52 (63%) patients were male. Among patients tested in serum and CSF (38/52), IgLON5-Abs were detected in both specimens (34/38, 89%), only in serum (3/38, 8%), and only in CSF (1/38, 3%; Table 1). The median time between symptom onset and diagnosis was 19 months (IQR 7–42; range 0.5–178); 32/52 patients (62%) had a chronic disease onset. CSF cell counts and protein levels were elevated in 5/45 patients (11%) and 24/43 patients (56%), respectively. CSF-restricted oligoclonal bands were found in 7/21 patients (33%). Brain MRI was abnormal in 17/50 patients (34%); T2 hyperintensities (9/50, 18%) and/or focal atrophy 7/50 (14%) were the most frequent abnormalities, mainly involving the brainstem, cerebellum, and/or cranial nerves (Supplementary Table 2, Fig. 1).

**Table 1:** General cohort data

Variable, *n*/*N* (%) or median (IQR)	*N* = 52
Age at diagnosis, years	72 (68–78)
Male sex	33 (52)
History of autoimmune disease	8/48 (17)
History of cancer^a^	10/48 (20)
Cancer detected after anti-IgLON5 disease diagnosis	
By whole-body CT scan	0/33 (0)
By whole-body FDG-PET scan^b^	1/21 (5)
Anti-IgLON5 antibodies	
Tested in serum and CSF	38/52 (73)
Positive in serum and CSF	34/38 (89)
Positive in CSF only	1/38 (3)
Positive in serum only	3/38 (8)
Tested in CSF only	11/52 (21)
Tested in serum only	3/52 (6)
Immune active treatments, *n*/*N* (%)	43/50 (86)
First-line treatments, *n*/*N* (%)	32/50 (64)
Time between symptom onset and first-line treatments, months	31 (11–48)
Corticosteroids, *n*/*N* (%)	18/50 (36)
Intravenous	16/18
Number of cycles	1 (1–3.5)
Oral	8/18
Immunoglobulins, *n*/*N* (%)	24/50 (48)
Number of cycles	2 (1–3)
Apheresis, *n*/*N* (%)	7/50 (14)
Second-line treatments, *n*/*N* (%)	35/50 (70)
Time between symptom onset and second-line treatments, months	25 (11–49)
Rituximab, *n*/*N* (%)	35/50 (70)
Number of cycles	2 (1–3)
Cyclophosphamide, *n*/*N* (%)	27/50 (54)
Number of cycles	7 (6–11)
Azathioprine, *n*/*N* (%)	3/50 (6)
Tocilizumab, *n*/*N* (%)	1/50 (2)

*CT* computerized tomography, *FDG-PET* fluorodeoxyglucose positron emission tomography

^a^Cancer types included breast carcinoma (*n* = 3), bladder urothelial carcinoma (*n* = 1), cutaneous melanoma (*n* = 1), meningioma (*n* = 1), small cell lung cancer (*n* = 1), prostate carcinoma (*n* = 1), sternal chondroma (*n* = 1), thymoma (*n* = 1)

^b^Lymphoma

### Application of the ICS to assess disease severity at diagnosis

After excluding two patients with > 3 missing symptoms, the ICS was scored at the time of diagnosis in 50/52 patients of the French cohort. All of them had symptoms in ≥ 2 ICS domains, including bulbar (88%), sleep (84%), movement disorders (90%), cognition (64%), and/or others (78%). Of note, the median number of ICS domains involved at diagnoses was 4 (IQR 2–5, range 2–5) and most patients had symptoms from 5 (22/50, 44%) or 4 (13/50, 26%) domains. The symptoms and their severity are detailed in Supplementary Table 1.

**Fig. 2:**
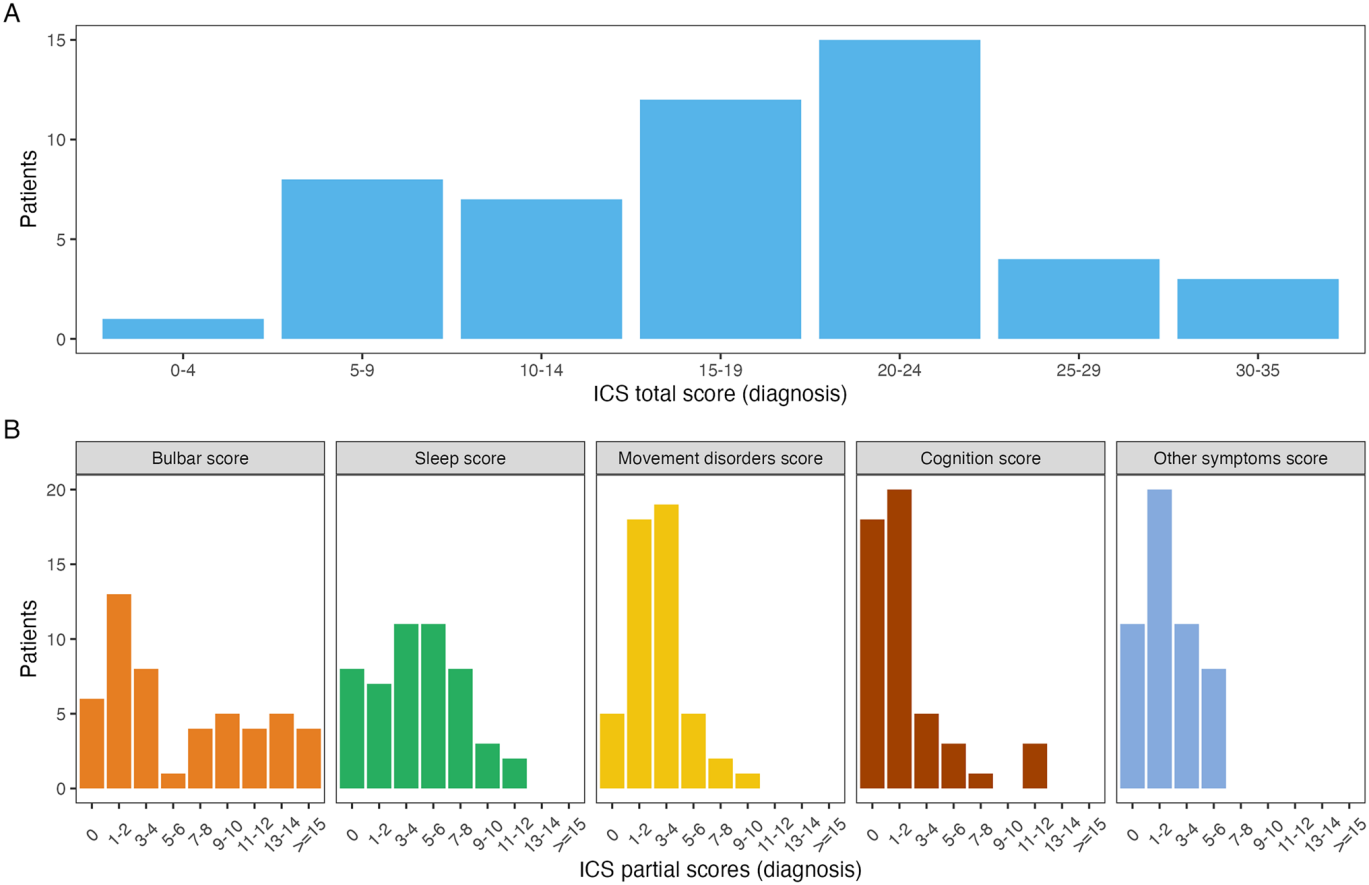
Total and partial anti-IgLON5 disease composite scores in the French cohort. Distribution of the total and partial composite anti-IgLON5 disease scores at diagnosis in the French cohort. *ICS* anti-IgLON5 composite score

The distribution of the total ICS in the French Cohort (median 18, IQR 12–23, range 4–35; Fig. 2) was similar to the Barcelona (median 18, range 2–31) and the GENERATE (median 12, range 4–23) cohorts [12]. However, the median partial ICS sleep and movement disorder scores were higher in the French (4, range 0–12 and 3, range 0–9, respectively) and the Barcelona (5, range 0–11 and 3, range 0–9, respectively) cohorts than in the GENERATE cohort (2, range 0–11 and 1, range 0–8, respectively), and the median partial ICS bulbar scores were higher in the French cohort (4, range 0–18) than in the Barcelona (3, range 0–14) and GENERATE (2.5, range 0–12) cohorts (Fig. 2; Table 2).

**Table 2:** Demographic, clinical, and severity data of patients with anti-IgLON5 disease

Variable, *N* (%) or median (range)	Cohorts			
	French (*N* = 50)	Barcelona and GENERATE (*N* = 86)	Barcelona (*N* = 46)	GENERATE (*N* = 40)
Age at diagnosis, years	72 (51–86)	66 (46–91)	66.5 (46–91)	64.5 (46–90)
Sex (male)	31 (62)	47 (55)	22 (48)	25 (62)
Time to diagnosis, months	21 (0.5–178)	25 (1–156)	23 (1–108)	31 (1–135)
Chronic presentation	31 (62)	60 (70)	30 (65)	30 (75)
mRS				
1	1 (2)	5 (6)	1 (2)	4 (10)
2	10 (20)	26 (30)	13 (28)	13 (33)
3	20 (40)	29 (34)	15 (33)	14 (35)
4	12 (24)	20 (23)	13 (28)	7 (18)
5	7 (14)	6 (7)	4 (9)	2 (5)
Total ICS	18 (4–35)	15 (2–31)	18 (2–31)	12 (4–23)
Partial scores				
Bulbar	4 (0–18)	3 (0–14)	3 (0–14)	2.5 (0–12)
Sleep	4 (0–12)	4 (0–11)	5 (0–11)	2 (0–11)
Movement disorders	3 (0–9)	2 (0–9)	3 (0–9)	1 (0–8)
Cognition	1 (0–12)	1 (0–12)	1 (0–12)	1 (0–6)
Other symptoms	2 (0–6)	2 (0–5)	2 (0–5)	2 (0–5)
Domains involved				
1	0	3 (3.5)	–	–
2	5 (10)	9 (10.5)	–	–
3	10 (20)	11 (13)	–	–
4	13 (26)	35 (41)	–	–
5	22 (44)	28 (33)	–	–

*ICS* anti-IgLON5 composite score, *mRS* modified Rankin Score

In the French cohort, the ICS was significantly correlated with the mRS at diagnosis (median mRS 3, IQR 3–4, range 1–5; rho 0.474, *p* < 0.001; Supplementary Fig. 2) and the time to diagnosis (rho 0.398, *p* = 0.004), but not with the age at diagnosis (rho 0.116, *p* = 0.423).

### Application of the ICS to assess clinical course

**Fig. 3:**
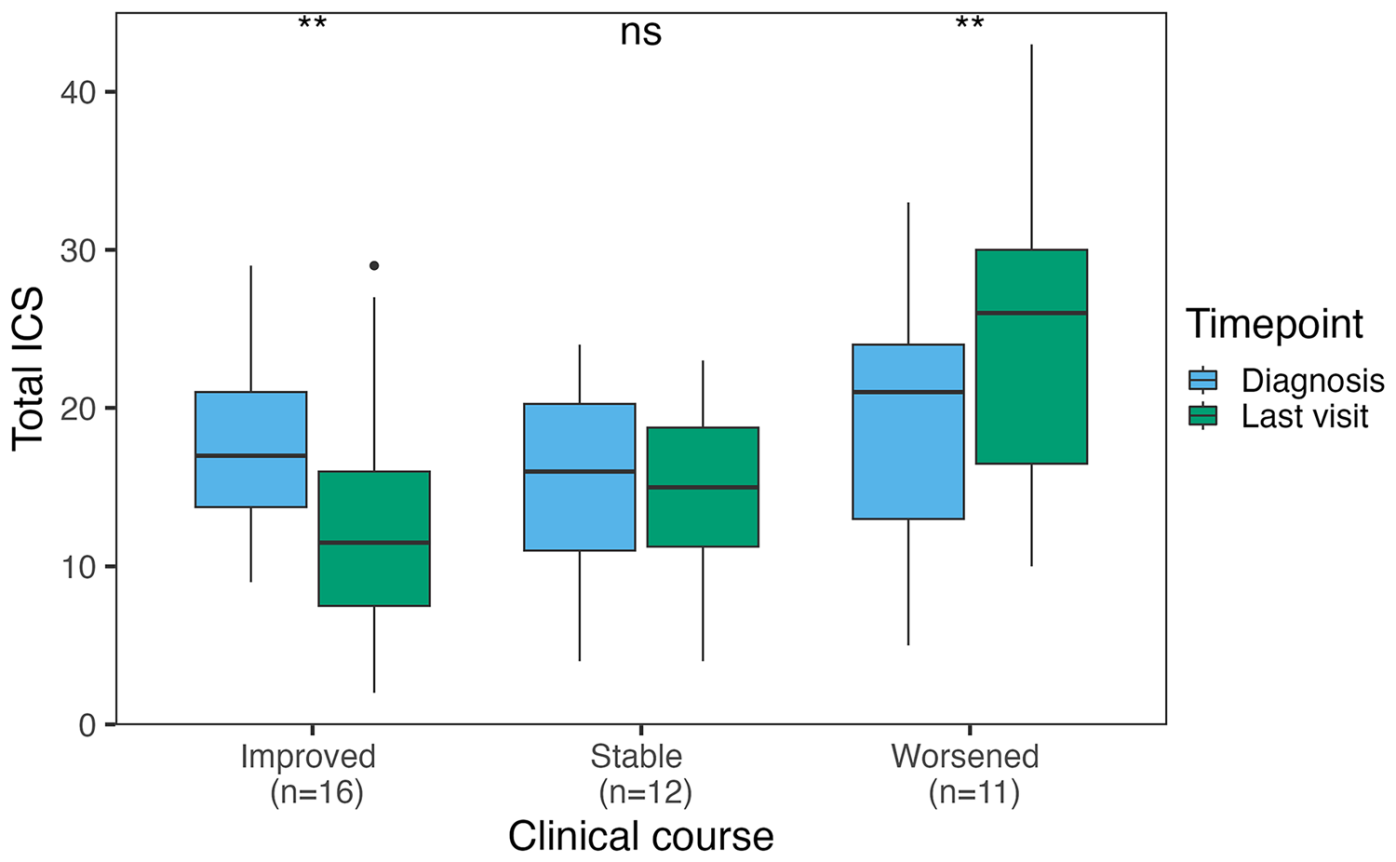
Total anti-IgLON5 disease composite score at diagnosis *versus* last visit. Distribution of the total anti-IgLON5 disease composite score at diagnosis and last visit in 39 patients with available follow-up information, classified according to clinical course (improved, stable, or worsened). *ns* non-significant; **p < = 0.01. *ICS* anti-IgLON5 composite score

**Fig. 4:**
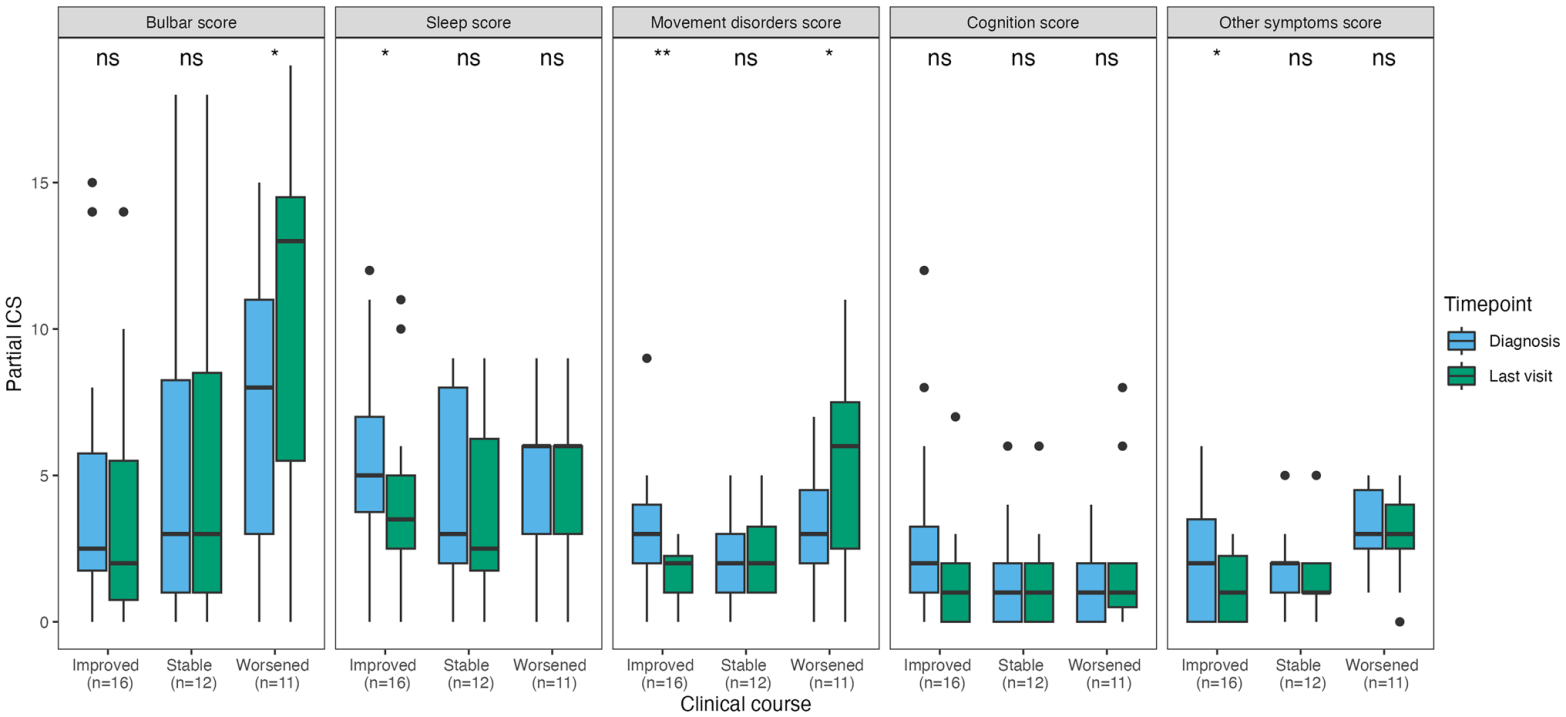
Partial anti-IgLON5 disease composite scores at diagnosis versus last visit. Distribution of the partial anti-IgLON5 disease composite (ICS) scores at diagnosis and last visit in 39 patients with available follow-up information, classified according to clinical course (improved, stable, or worsened). *ns* non-significant; *p < = 0.05; **p < = 0.01. *ICS* anti-IgLON5 composite score

Clinical course was assessed in 46/52 patients with follow-up information. Among them, 7/46 (16%) died shortly (median 0.5 months, IQR 0–1) after diagnosis (6/7 because of anti-IgLON5 disease and 1/7 due to septic shock), of whom 3 had received first-line treatments and none had received second-line treatments. Their median ICS at diagnosis (scored in 5/7 because the remaining 2 had > 3 missing symptoms) was 24 (range 17–35). Among the remaining 39 patients, 16/39 (35%) improved, 12/39 (26%) were clinically stable, and 11/39 (24%) worsened during follow-up. In patients who improved, at last visit (median 23 months after diagnosis, IQR 10–43, range 4–83) the total ICS was lower (median 12, IQR 8–16, range 2–29) than at diagnosis (median 17, IQR 14–21, range 9–29, *p* = 0.004). However, 3 of these 16 patients (19%) experienced irreversible clinical deterioration after initial clinical improvement; their median ICS first improved from 20 (IQR 18–24) to 10 (IQR 8–13), then worsened to 27 (IQR 20–28) at last visit. In patients who were stable, at last visit (median 17 months after diagnosis, IQR 11–26, range 5–27), the ICS was similar (median 15, IQR 11–19, range 4–23) to that at diagnosis (median 16, IQR 11–20, range 4–24, *p* = 0.222). In patients who worsened, at last visit (median 12 months after diagnosis, IQR 9–16, range 7–50), the ICS was higher compared to that at diagnosis (median 26, IQR 16–30, range 10–43 vs median 21, IQR 13–24, range 5–33, *p* = 0.006; Fig. 3). Of note, in patients who improved, only the partial ICS scores for sleep, movement disorders, and other symptoms were significantly lower at last visit compared with those at diagnosis, while in patients who worsened, only the partial scores for bulbar and movement disorders were significantly higher at last visit compared to those at diagnosis (Fig. 4). No significant association was found between demographic features, clinical onset pattern, total and partial ICS scores at diagnosis, CSF and MRI findings, or treatment variables (including the frequency and the time to first and second-line immunotherapy) and the clinical course (improving, stable, worsening, fulminant; Supplementary Table 3). The odds of an improved or stable clinical course, compared to a worsening course, were significantly higher in patients in whom second-line treatments were administered within the first year of clinical onset (OR 1.44, 95% CI [1.04; 1.99]; *p* = 0.032), while the type of clinical course was not significantly affected by the administration of first-line treatments within the first year of clinical onset (OR 1.15, 95% CI [0.77; 1.71]; *p* = 0.508).

### Application of the ICS to predict the risk of mortality

The median follow-up of the French cohort was 11 months (IQR 4–24, range 0–87). By the end of the study period, 20 patients had died (38%) in a median of 7 months (IQR 2–14, range 0–87) from diagnosis (Supplementary Fig. 3). The cause of death was reported in 15 patients: it was attributed to the neurological disease (respiratory failure in all) in 12/15 and to other causes in 3/15 (sepsis, *n* = 2; stroke, *n* = 1).

**Fig. 5:**
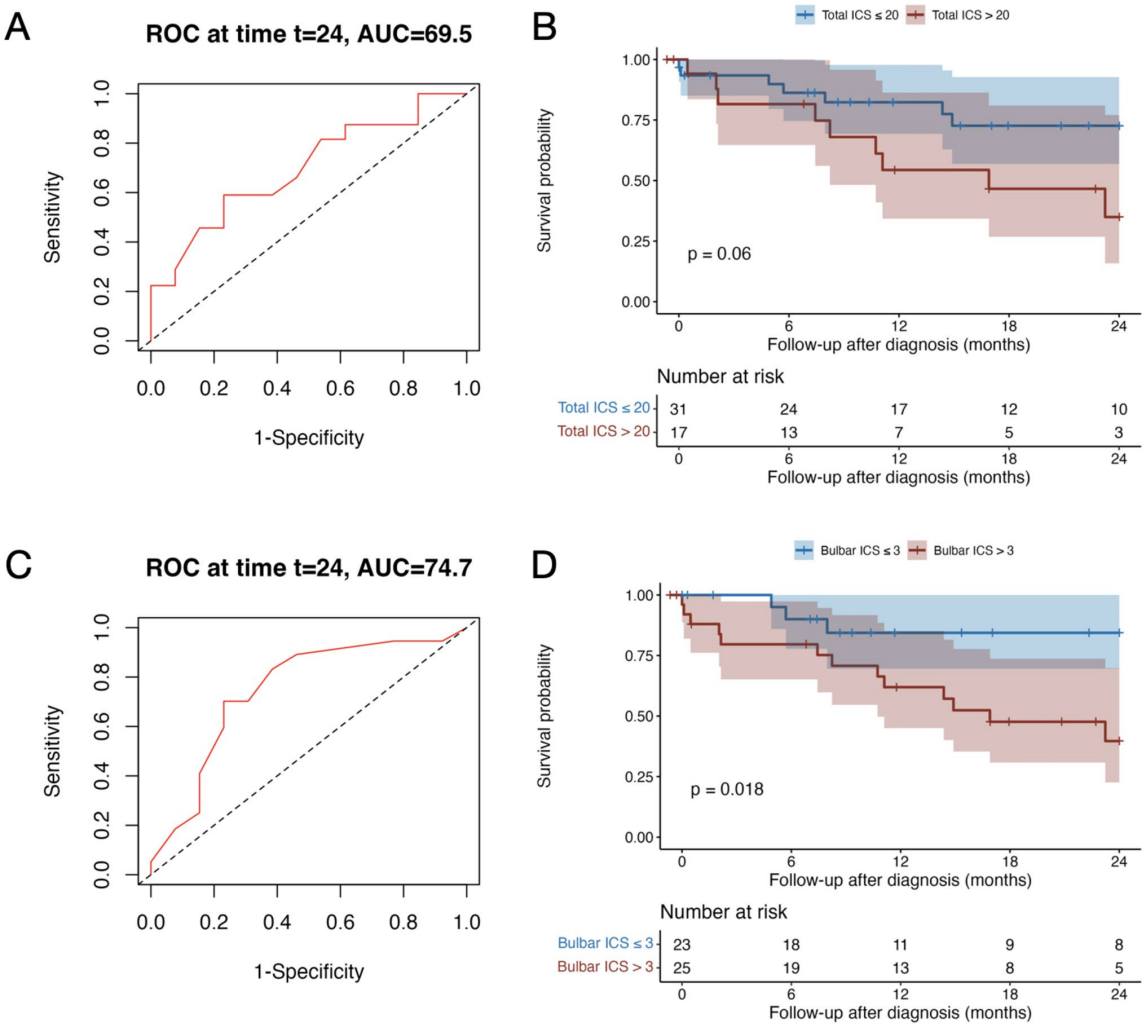
Total anti-IgLON5 disease composite score and partial bulbar score at diagnosis and 2-year mortality. Receiver operating characteristic (ROC) curves of total ICS to discriminate 2-year mortality (**A**). Kaplan–Meier and comparison by log-rank test of the 2-year survival between patients with total ICS at diagnosis ≤ 20 and those with total ICS at diagnosis > 20. Tick marks indicate censored patients (**B**). ROC curves of partial bulbar ICS to discriminate 2-year mortality (**C**). Kaplan–Meyer and comparison by log-rank test of the 2-year survival between patients with bulbar ICS at diagnosis ≤ 3 and those with bulbar ICS at diagnosis > 3. Tick marks indicate censored patients (**D**). *AUC* area under the curve, *ICS* anti-IgLON5 composite score, *ROC* receiver operating characteristic

In the ROC analyses, the total ICS at diagnosis significantly predicted the 2-year mortality (AUC 69.51, 95% CI [50.19; 88.83]) with an optimal cut-off > 20 (sensitivity 59%, specificity 77%; Fig. 5A). In addition, the partial bulbar score at diagnosis significantly predicted the 2-year mortality (AUC 74.68, 95% CI [56.17; 93.19]) with an optimal cut-off > 3 (sensitivity 83%, specificity 62%; Fig. 5C); none of the other ICS partial scores significantly predicted the 2-year mortality. The 2-year survival probability was significantly lower in patients with a bulbar partial score > 3 (*p* = 0.018; Fig. 5D) and tended to be lower in patients with a total ICS > 20 (*p* = 0.06; Fig. 5B). In the Cox regression analysis, a bulbar score > 3 was also significantly associated with an increased 2-year mortality risk, independently of the age (*p* = 0.035; HR 3.94, 95% CI [1.10; 14.07]), while the effect of the total ICS on this risk was not significant (*p* = 0.097; Supplementary Fig. 4A, B).

## Discussion

The results of the present study underscore the reproducibility and clinical utility of the ICS in assessing severity and clinical course of the anti-IgLON5 disease. The findings also highlight that the total ICS score at diagnosis and, especially the partial bulbar score, may predict the risk of mortality.

In this French cohort, by the time of diagnosis, all patients had a multidomain clinical presentation, a similar figure than in the ICS development cohorts [12], with a predominance of bulbar, sleep, and movement disorders, which is consistent with the classical clinical profile of anti-IgLON5 disease [2, 8, 10, 11]. Likewise, other clinical and paraclinical findings were similar to those of previously published cohorts, such as the long time to diagnosis, the more frequent chronic onset, and the frequent lack of inflammatory changes in CSF and brain MRI [2, 8, 10, 11]. Reassuringly, the ICS at diagnosis had a similar distribution in three independent nationwide cohorts; in all of them, the ICS was correlated with the mRS and its reassessment during follow-up was able to capture the clinical course (improvement, stability, or worsening) [12]. These findings highlight the value of the ICS as a reproducible tool to assess severity and monitor the clinical course of anti-IgLON5 disease. Of note, in contrast to what was reported for the German and the Spanish cohorts, the total ICS also showed a significant correlation with the time to diagnosis among French patients, which is consistent with the symptom progression and disability accumulation over time, likely related to the spreading of neurodegeneration in the late stages of the disease [7].

Furthermore, a critical finding of the present study is that the 2-year mortality risk was significantly higher in patients with a total ICS > 20 at diagnosis. It was even more affected by a partial bulbar score > 3 but was not affected by the other partial ICS scores (including sleep, movement disorders, and cognition). This result might explain the higher mortality in the French cohort compared with the German cohort (~ 40% vs ~ 20%), as the median bulbar score was higher in the former than in the latter [8]. Bulbar dysfunction is a main feature of anti-IgLON5 disease [8, 10, 17], and its negative impact on survival has previously been demonstrated in other neurological disorders, including anti-IgLON5 disease mimics, such as amyotrophic lateral sclerosis [18] and myasthenia gravis [19]. The impact of bulbar dysfunction on the mortality risk of anti-IgLON5 disease is explained by the high frequency of severe dysphagia (frequently leading to aspiration pneumonia), central hypoventilation, and/or laryngeal occlusion, all causing respiratory failure, which represented the main cause of death in the present and previously published cohorts [8, 10]. Apart from predicting mortality, the partial ICS bulbar score at diagnosis also tended to be associated, in the present study, with a worsening clinical course, and did not significantly decrease even in patients who showed an overall improvement during follow-up. Altogether, these findings highlight the need for a careful screening and intensive management of bulbar dysfunction in patients with anti-IgLON5 disease, by providing supportive treatments (e.g. elective tracheostomy, artificial nutrition) and anticipating aggressive immune-active therapies (e.g. rituximab and/or cyclophosphamide). Notably, even though in the present and previous studies, only a minority of patients with anti-IgLON5 disease improved after immunotherapy [2, 8, 10, 11, 17], we and others [8] found that the administration of long-acting immunotherapies (mostly rituximab and/or cyclophosphamide) within the first year of clinical onset was associated with a better prognosis, corroborating the hypothesis of an inflammatory time window susceptible to immunotherapy response followed by a neurodegeneration phase when immunotherapies have no effect [2, 7, 8].

A few study limitations need to be acknowledged. First, some ICS symptoms might have been inaccurately scored due to the retrospective data collection. Second, the classification of the clinical course was limited by the heterogeneity in the timing of visits and the duration of follow-up. Additionally, the use of various immune-active treatments in different combinations prevented to assess the response to individual drugs. Finally, the cause of death was not reported in a substantial number of patients, and the ROC and survival analyses need to be interpreted with caution due to the relatively low number of patients included. Therefore, prospective, multicenter studies, enrolling a larger number of patients, are needed to overcome the study limitations and confirm its findings.

In conclusion, the ICS is a valid and reproducible tool to assess the severity and clinical course of anti-IgLON5 disease, which encourages the use of this score in both the research and clinical contexts. Patients with a bulbar score > 3 at diagnosis are at increased risk of mortality.

## Supplementary Information

Below is the link to the electronic supplementary material.

Supplementary file1 (DOCX 3693 kb)

## Data Availability

Data reported in this study are available within the article and/or its supplementary material. More information regarding the data will be shared by the corresponding author on reasonable request.
