# Supplementary material for: Application of the anti-IgLON5 disease composite score to assess severity, clinical course, and mortality in a French cohort
Source: J Neurol. 2025 Mar 19;272(4):273. doi: 10.1007/s00415-025-13001-7 (PMC11922985; doi:10.1007/s00415-025-13001-7)
Supplement: Supplementary file 1 — Supplementary file1 (DOCX 3693 kb) [file 415_2025_13001_MOESM1_ESM.docx]

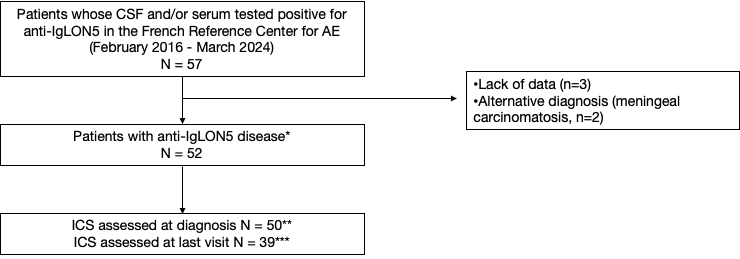


**Supplementary Figure 1. Study flow chart**

*In 2 patients the diagnosis was retrospective (post-mortem in 1/2), as the IgLON5-specific cell-based assay was performed on samples with a diffuse neuropilar staining collected before the first description of IgLON5-Abs. Two patients had coexisting neural antibodies (LGI1, n=1; GAD65, n=1).

**Anti-IgLON5 disease composite score at diagnosis was not scored in 2/52 patients with >3 missing symptoms.^12^

***Anti-IgLON5 disease composite score at last visit was not scored in 7/52 patients who died shortly after diagnosis (fulminant course) and 6/52 patients without follow-up information.

Abbreviations: AE=autoimmune encephalitis; CSF=cerebrospinal fluid; ICS= anti-IgLON5 disease composite score

^
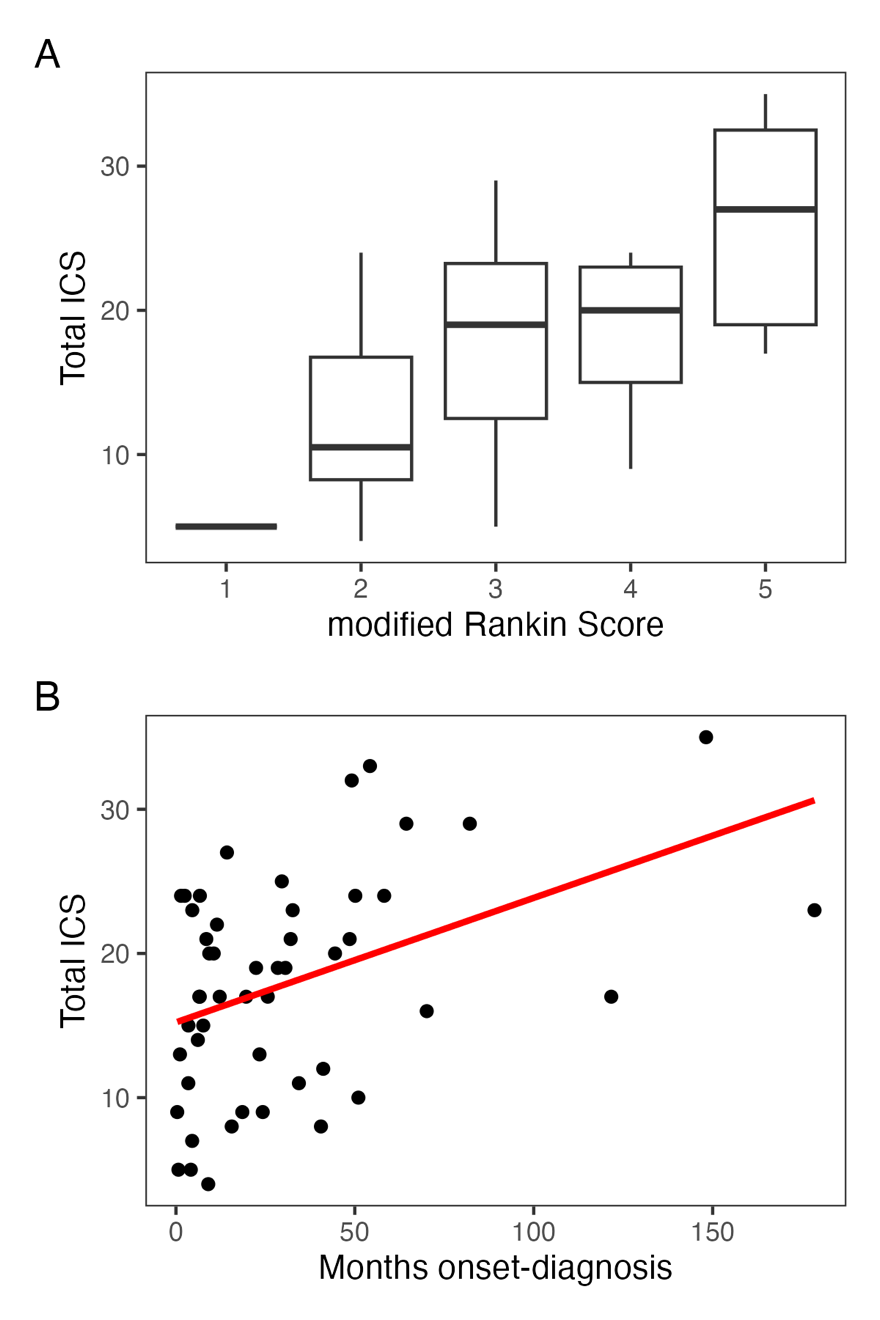
^

**Supplementary Figure 2. Anti-IgLON5 disease composite score correlations in the French cohort**

A) Correlation between the anti-IgLON5 disease composite total score (ICS) and modified Rankin Score (mRS) at diagnosis. B) Correlation between the ICS at diagnosis and the time to diagnosis (months).

Abbreviations: ICS=anti-IgLON5 composite score.

**
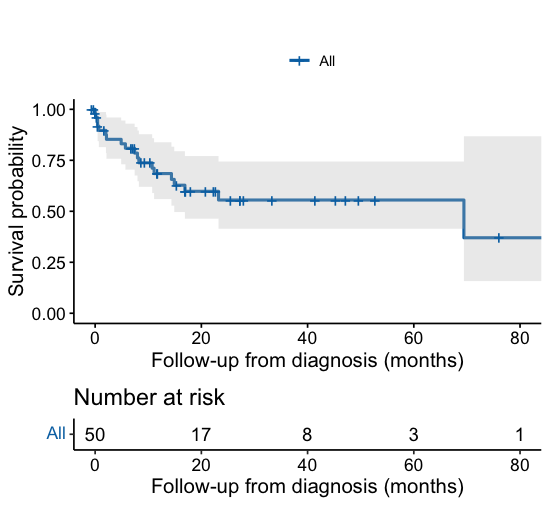
**

**Supplementary Figure 3. Probability of survival in the anti-IgLON5 disease French cohort**

Kaplan–Meier curve. Tick marks indicate censored patients.

**
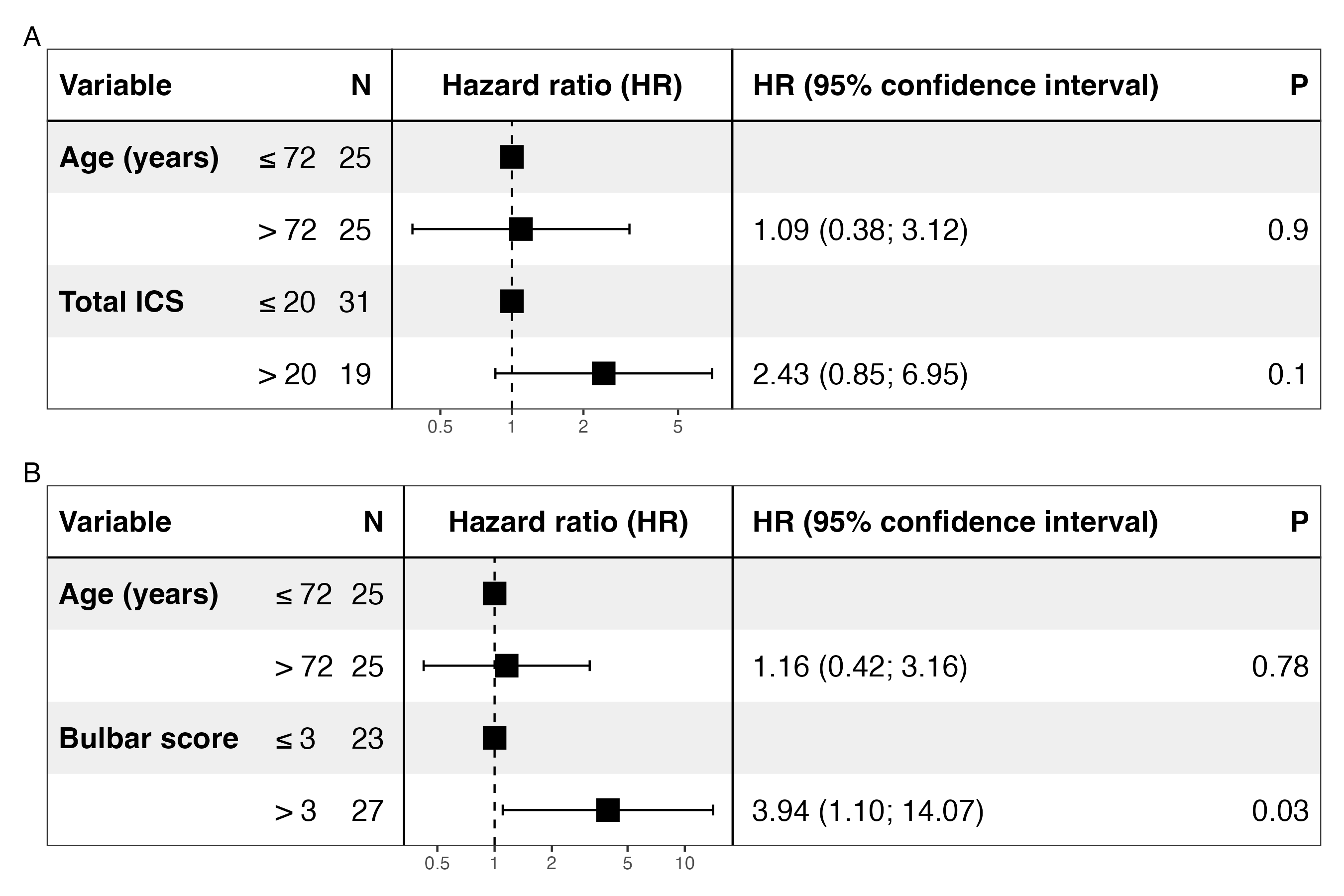
**

**Supplementary Figure 4. Total anti-IgLON5 disease composite score and partial bulbar score and 2-year mortality: multivariable analysis**

A) Forest plot of multivariable Cox analysis evaluating the age-adjusted effect of the total ICS on 2-year mortality. B) Forest plot of multivariable Cox analysis evaluating the age-adjusted effect of the partial bulbar ICS on 2-year mortality.

Abbreviations: ICS=anti-IgLON5 composite score.

**Supplementary Table 1. Symptom frequency and severity at diagnosis in the French anti-IgLON5 disease cohort**

| **Variable** | **Total number of patients = 50**  **N (%)** |
| --- | --- |
| Bulbar symptoms | 44 (88) |
| Stridor | 16 (32) |
| Mild (1) | 4 (8) |
| Moderate (2) | 1 (2) |
| Severe (6) | 11 (22) |
| Central hypoventilation | 18 (37) |
| Mild (1) | 2 (4) |
| Moderate (2) | 7 (14) |
| Severe (6) | 9 (18) |
| Unknown | 1 |
| Dysphagia | 40 (80) |
| Mild (1) | 14 (28) |
| Moderate (2) | 9 (18) |
| Severe (6) | 17 (34) |
| Dysarthria | 24 (48) |
| Mild (1) | 16 (32) |
| Moderate (2) | 4 (8) |
| Severe (3) | 4 (8) |
| Sleep disorders | 42 (84) |
| Abnormal movements/behaviors | 22 (45) |
| Mild (1) | 1 (2) |
| Moderate (2) | 15 (31) |
| Severe (3) | 6 (12) |
| Unknown | 1 |
| Insomnia | 25 (50) |
| Mild (1) | 7 (14) |
| Moderate (2) | 12 (24) |
| Severe (3) | 6 (12) |
| Excessive daytime sleepiness | 21 (42) |
| Mild (1) | 8 (16) |
| Moderate (2) | 8 (16) |
| Severe (3) | 5 (10) |
| Obstructive sleep apnea | 35 (71) |
| Mild (1) | 8 (16) |
| Moderate (2) | 6 (12) |
| Severe (3) | 21 (43) |
| Unknown | 1 |
| Movement disorders | 45 (90) |
| Gait difficulties and falls | 41 (84) |
| Mild (1) | 21 (43) |
| Moderate (2) | 18 (37) |
| Severe (6) | 2 (4) |
| Unknown | 1 |
| Chorea | 9 (18) |
| Mild (1) | 5 (10) |
| Moderate (2) | 4 (8) |
| Orofacial dyskinesia | 12 (24) |
| Mild (1) | 6 (12) |
| Moderate (2) | 2 (4) |
| Severe (3) | 4 (8) |
| Other movement disorders* | 13 (47) |
| Mild (1) | 8 (16) |
| Moderate (2) | 13 (27) |
| Severe (3) | 2 (4) |
| Unknown | 1 |
| Cognition | 32 (64) |
| Cognitive impairment | 31 (63) |
| Mild (1) | 10 (20) |
| Moderate (2) | 15 (31) |
| Severe (6) | 6 (12) |
| Unknown | 1 |
| Neuropsychiatric (psychosis, delirium) | 11 (22) |
| Mild (1) | 2 (4) |
| Moderate (2) | 5 (10) |
| Severe (6) | 4 (8) |
| Other symptoms | 39 (78) |
| Oculomotor abnormalities | 27 (56) |
| Mild (1) | 10 (21) |
| Moderate (2) | 13 (27) |
| Severe (3) | 4 (8) |
| Unknown | 2 |
| Dysautonomia | 22 (44) |
| Mild (1) | 12 (24) |
| Moderate (2) | 9 (18) |
| Severe (3) | 1 (2) |
| Fasciculations | 14 (28) |
| Mild (1) | 3 (6) |
| Moderate (2) | 8 (16) |
| Severe (3) | 3 (6) |

*Myoclonus (n=11), dystonia (n=7), tremor (n=7), and/or not otherwise specified (n=2).

Symptom severity was classified as mild (1), moderate (2), or severe (3 or 6) according to the anti-IgLON5 composite score guidelines provided by Gaig *et al.* [12].

**Supplementary Table 2. Brain MRI findings**

| **Variable, n/N (%)** | **N = 52** |
| --- | --- |
| Brain MRI |  |
| Normal or non-specific | 33/50 (66) |
| Leukoaraiosis | 15 |
| Cortico-subcortical atrophy | 7 |
| Abnormal | 17/50 (34) |
| Focal atrophy | 7/50 (14) |
| Brainstem | 4 |
| Cerebellar | 3 |
| Temporomesial | 1 |
| Basal ganglia | 1 |
| T2/FLAIR hyperintensity | 9/50 (18) |
| Cranial nerves* | 3 |
| Temporomesial | 2 |
| Brainstem | 2 |
| Pyramidal tracts | 2 |
| Basal ganglia | 2 |
| Temporopolar | 1 |
| Thalamic | 1 |
| Other abnormalities | 4/50 (8) |
| Cranial nerve contrast enhancement** | 1 |
| Cortical DWI hyperintesity | 1 |
| T2* hypointensity in lenticular nuclei | 1 |
| T2* lobar hypointensity and cortical siderosis | 1 |
| Hyperintensity of lateral pterygoid and temporal muscles | 1 |

*Trigeminal nerves in 2/3 patients.

** Facial nerves (1/1)

Abbreviations: DWI=Diffusion-weighted imaging cortical hyperintensity; FLAIR= Fluid attenuated inversion recovery.

**Supplementary Table 3. Comparison between patients with different disease courses**

| **Variable, N (%) or median (IQR)** | **Improved N = 16** | **Stable N = 12** | **Worsened N = 11** | **Fulminant N = 7** | **p-value** | **q-value**** |
| --- | --- | --- | --- | --- | --- | --- |
| Age at diagnosis, years | 69 (64-71) | 76 (73-81) | 71 (64-78) | 77 (69-80) | 0.034 | 0.125 |
| Male sex | 9 (56%) | 7 (58%) | 6 (55%) | 7 (100%) | 0.160 | 0.251 |
| Time to diagnosis since clinical onset, months | 17 (6-37) | 10 (8-27) | 49 (27-53) | 12 (4-32) | 0.097 | 0.213 |
| Clinical onset pattern |  |  |  |  | >0.999 | >0.999 |
| Chronic | 10 (62%) | 8 (67%) | 7 (64%) | 4 (57%) |  |  |
| Subacute | 6 (38%) | 4 (33%) | 4 (36%) | 3 (43%) |  |  |
| CSF pleocytosis | 1 (6.7%) | 2 (18%) | 2 (20%) | 0 (0%) | 0.620 | 0.682 |
| Unknown | 1 | 1 | 1 | 1 |  |  |
| CSF proteins >0.45 g/L | 12 (80%) | 6 (60%) | 7 (70%) | 1 (20%) | 0.108 | 0.216 |
| Immune active treatments | 14 (88%) | 12 (100%) | 11 (100%) | 3 (43%) | 0.325* | 0.624* |
| First-line | 10 (62%) | 8 (67%) | 8 (73%) | 3 (43%) | 0.912* | >0.999* |
| Second-line | 14 (88%) | 12 (100%) | 9 (82%) | 0 (0%) | 0.360* | 0.624* |
| Time to first line since clinical onset, months | 29 (14-43) | 21 (7-36) | 40 (29-49) | 49 (31-85) | 0.416* | 0.624* |
| Unknown | 6 | 4 | 3 | 4 |  |  |
| Time to second-line since clinical onset, months | 21 (9-37) | 13 (10-28) | 50 (26-55) | - | 0.041* | 0.177* |
| Unknown | 3 | 0 | 2 | 7 |  |  |
| Total ICS at diagnosis | 17 (14-21) | 16 (11-20) | 21 (13-24) | 24 (17-24) | 0.245 | 0.355 |
| Unknown | 0 | 0 | 0 | 2 |  |  |
| Bulbar score | 2.5 (1.8-5.8) | 3.0 (1.0-8.2) | 8.0 (3.0- 11.0) | 12.0 (12.0- 12.0) | 0.066 | 0.160 |
| Unknown | 0 | 0 | 0 | 2 |  |  |
| Sleep score | 5.00 (3.75-7.00) | 3.00 (2.00-8.00) | 6.00 (3.00-6.00) | 0.00 (0.00-3.00) | 0.121 | 0.222 |
| Unknown | 0 | 0 | 0 | 2 |  |  |
| Movement disorder score | 3.00 (2.00-4.00) | 2.00 (1.00-3.00) | 3.00 (2.00-4.50) | 2.00 (1.00-4.00) | 0.523 | 0.606 |
| Unknown | 0 | 0 | 0 | 2 |  |  |
| Cognition score | 2.0 (1.0-3.2) | 1.0 (0.0-2.0) | 1.0 (0.0-2.0) | 4.0 (0.0-12.0) | 0.275 | 0.355 |
| Unknown | 0 | 0 | 0 | 2 |  |  |
| Other score | 2.00 (0.00-3.50) | 2.00 (1.00-2.00) | 3.00 (2.50-4.50) | 0.00 (0.00-0.00) | 0.049 | 0.134 |
| Unknown | 0 | 0 | 0 | 2 |  |  |
| Follow-up duration since diagnosis (months) | 23 (10-43) | 17 (11-26) | 12 (9-16) | 0 (0-1) | <0.001 | 0.003 |
| Total ICS at last visit | 12 (8-16) | 15 (11-19) | 26 (16-30) | - | 0.006 | 0.065 |
| N | 16 | 12 | 11 | 0 |  |  |
| Unknown | 0 | 0 | 0 | 7 |  |  |

*The patients with a fulminant course were excluded from the comparison.

**False discovery rate correction for multiple testing.

Abbreviations: CSF=cerebrospinal fluid; ICS=Anti-IgLON5 disease composite score.
